# Antimicrobial use in European acute care hospitals: results from the second point prevalence survey (PPS) of healthcare-associated infections and antimicrobial use, 2016 to 2017

**DOI:** 10.2807/1560-7917.ES.23.46.1800393

**Published:** 2018-11-15

**Authors:** Diamantis Plachouras, Tommi Kärki, Sonja Hansen, Susan Hopkins, Outi Lyytikäinen, Maria Luisa Moro, Jacqui Reilly, Peter Zarb, Walter Zingg, Pete Kinross, Klaus Weist, Dominique L Monnet, Carl Suetens, the Point Prevalence Survey Study Group

**Affiliations:** 1 European Centre for Disease Prevention and Control, Stockholm, Sweden; 2 Institute of Hygiene and Environmental Medicine, Charité – University Medicine Berlin, Berlin, Germany; 3 Public Health England, London, United Kingdom; 4 National Institute for Health and Welfare (THL), Department of Health Security, Helsinki, Finland; 5 Agenzia sanitaria e sociale regionale – Regione Emilia Romagna, Bologna, Italy; 6 National Services Scotland, Health Protection Scotland, Glasgow, United Kingdom; 7 Glasgow Caledonian University, Glasgow, United Kingdom; 8 Mater Dei Hospital, Msida, Malta; 9 Imperial College, London, United Kingdom; 10 Members of the Point Prevalence Survey Study Group are listed at the end of this article

**Keywords:** antimicrobial use, point-prevalence survey, hospitals, surveillance, healthcare-associated infections, antibiotic use

## Abstract

Antimicrobial agents used to treat infections are life-saving. Overuse may result in more frequent adverse effects and emergence of multidrug-resistant microorganisms. In 2016–17, we performed the second point-prevalence survey (PPS) of healthcare-associated infections (HAIs) and antimicrobial use in European acute care hospitals. We included 1,209 hospitals and 310,755 patients in 28 of 31 European Union/European Economic Area (EU/EEA) countries. The weighted prevalence of antimicrobial use in the EU/EEA was 30.5% (95% CI: 29.2–31.9%). The most common indication for prescribing antimicrobials was treatment of a community-acquired infection, followed by treatment of HAI and surgical prophylaxis. Over half (54.2%) of antimicrobials for surgical prophylaxis were prescribed for more than 1 day. The most common infections treated by antimicrobials were respiratory tract infections and the most commonly prescribed antimicrobial agents were penicillins with beta-lactamase inhibitors. There was wide variation of patients on antimicrobials, in the selection of antimicrobial agents and in antimicrobial stewardship resources and activities across the participating countries. The results of the PPS provide detailed information on antimicrobial use in European acute care hospitals, enable comparisons between countries and hospitals, and highlight key areas for national and European action that will support efforts towards prudent use of antimicrobials.

## Background

Antimicrobials are commonly used in acute care hospitals for the treatment of both community-acquired and healthcare-associated infections (HAIs), and for surgical prophylaxis [1]. Studies have indicated that some antimicrobial use may be unnecessary and in instances when use is required, the selection, dose, route of administration and duration of treatment may be inappropriate [2,3]. Through selection pressure, antimicrobials contribute to the emergence and spread of antimicrobial resistance (AMR) [4]. Moreover, antimicrobial use has adverse consequences, including HAIs caused by *Clostridium difficile* [5,6], multidrug-resistant organisms [7] and fungi [8].

Data on antimicrobial consumption in acute care hospitals are necessary to assess the magnitude, the reasons and determinants of antimicrobial use and to inform public health policies that are promoting prudent use of antimicrobials. In June 2017, the European Commission published the European guidelines for the prudent use of antimicrobials in human medicine [9]. These guidelines recommend establishing antimicrobial stewardship programmes in all healthcare facilities. Although antimicrobial consumption in hospitals is measured at a national level by some EU/EEA countries, methodologies are not always consistent between countries and therefore preclude valid comparisons. The European Surveillance of Antimicrobial Consumption Network (ESAC-Net) monitors the use of antimicrobials in the EU/EEA, but does not provide uniform information on antimicrobial use in hospitals and does not include clinical data to assess the appropriateness of antimicrobial prescriptions [10].

Point prevalence surveys (PPSs) are a feasible method to assess antimicrobial use in hospitals, and their value in identifying targets for interventions has been demonstrated [2,11]. The European Centre for Disease Prevention and Control (ECDC) PPS of HAIs and antimicrobial use in European acute care hospitals applies a standardised methodology for the estimation of the prevalence of both HAIs and antimicrobial use across the EU/EEA. The first ECDC PPS in 2011–12 indicated that 32.7% of patients in acute care hospitals received one or more antimicrobial agents on the day of the survey, which translated to more than 450,000 patients receiving at least one antimicrobial agent on any given day in European acute care hospitals [1].

In this study, based on data from the second PPS in 2016–17, we aimed at estimating the prevalence of antimicrobial use and describing the indications and the prescribed antimicrobial agents. Further, we aimed to raise awareness, identify targets for improvement and provide a standardised tool for evaluating the effect of local, regional and national policies on strengthening prudent use of antimicrobials in European acute care hospitals.

## Methods

### Survey design

The PPS was performed in 28 EU/EEA countries and one EU candidate country, Serbia. The countries were recommended to select the participating acute care hospitals by systematic random sampling. Data were collected by trained staff on 1 day per ward during four possible periods in 2016–17. The periods were selected to be out of the winter period (December–February) when antimicrobial use is the highest and out of the summer holiday season (July–August) when staffing at hospitals is usually low.

All participating countries applied a standardised protocol updated from a version used in an earlier PPS conducted in 2011–12 [12]; the main update was the addition of a larger number of structure and process indicators for the prevention of HAIs and for antimicrobial stewardship. All patients admitted to the ward before or at 0800 on the day of the PPS and were still present at the time of the PPS were included. It was also possible to provide aggregated denominator data at ward level (‘light’ protocol).

### Data collection

Data collected included; hospital type and size, ward specialty, patient demographic data and risk factors and whether the patient was receiving one or more antimicrobial agent at the time of the PPS.

For patients receiving one or more antimicrobials additional data were collected for each antimicrobial prescribed including; the agent, the route of administration, the dosage and indication based on prescriber judgement (treatment of community, hospital or long-term care acquired infection, surgical or medical prophylaxis), diagnosis by anatomical site in case of treatment (e.g. pneumonia, urinary tract infection etc.), documentation of the reason for antimicrobial prescription in the medical records, and whether the current antimicrobial regimen was the same as the one that had been initiated. In case of change, the reason for change had to be indicated (escalation, de-escalation, switch from intravenous to oral, adverse effects, other or unknown).

#### Prevalence of antimicrobial use and the number of Defined Daily Doses

The 2018 version of the Anatomical Therapeutic Chemical/Defined Daily Dose (ATC/DDD) index of the World Health Organization (WHO) Collaborating Centre for Drug Statistics Methodology was used for calculating the prevalence of antimicrobial use and the number of DDDs per 100 patients on the day of PPS [13]. Antimicrobial agents for systemic use within ATC groups A07AA (intestinal antiinfectives), D01BA (dermatological antifungals for systemic use), J01 (antibacterials for systemic use), J02 (antimycotics for systemic use), J04 (antimycobacterials) as second-line treatment of e.g. meticillin-resistant *Staphylococcus aureus* (MRSA) infections (rifampicin) or for treatment of mycobacteria other than tuberculosis (MOTT) and P01AB (nitroimidazole-derived antiprotozoals) were included. Antiviral agents and antimicrobials for the treatment of mycobacteria were not included. For the calculation of the number of DDD per 100 patients, children and adolescents (< 18 years of age) and neonates were excluded, as DDDs are defined for adults only.

#### Structure and process indicators

Data on the structure and process indicators in relation to antimicrobial stewardship were collected at hospital level including; number of full-time equivalent antimicrobial stewardship consultants, existence of a formal hospital procedure for post-prescription review of the appropriateness of an antimicrobial within 72 hours (3 calendar days) from the initial order and participation in a national or regional hospital antimicrobial consumption surveillance network.

Data from the United Kingdom (UK) were reported separately for the four administrations: UK-England, UK-Northern Ireland, UK-Scotland and UK-Wales.

### Descriptive analysis

All analyses were performed with R, version 3.4.0 (R Foundation for Statistical Computing, Vienna, Austria).

Country representativeness of the sample was considered ‘optimal’ if the recommended systematic random sampling of hospitals was used, ‘good’ if a sufficient number of representative hospitals was selected applying a different methodology or ‘poor’ if there was no systematic selection of a representative sample hospitals. For countries contributing to the survey with more than 20,000 patients, a randomised sub-sample was used in the final analysis to avoid over-representation of these countries when making analyses for the EU/EEA overall.

The prevalence of antimicrobial use was reported as the percentage of patients receiving at least one antimicrobial agent on the day of the survey. Antimicrobial groups and agents were classified according to the ATC/DDD index at the level of the chemical group (4^th^ ATC level) and the chemical substance (5^th^ ATC level). The relative frequencies of antimicrobial groups (4^th^ ATC level) were calculated. In addition, the relative frequencies of individual antimicrobial agents (5^th^ ATC level) that represented the Drug Utilisation 75% (DU75%), i.e. describing the agents that made 75% of total antimicrobial use in the participating hospitals, were also reported [14].

The proportion of the broad-spectrum antibacterials, among all antibacterials for systemic use (ATC J01), was also calculated – as proposed in the ECDC, European Food Safety Authority (EFSA) and European Medicines Agency (EMA) Joint Scientific Opinion on a list of outcome indicators for surveillance of AMR and antimicrobial consumption in humans and food producing animals [15]. The following antimicrobial groups and agents were included under broad-spectrum antimicrobials: piperacillin and beta-lactamase inhibitor (ATC J01CR05), third- and fourth-generation cephalosporins (J01DD and J01DE), monobactams (J01DF), carbapenems (J01DH), fluoroquinolones (J01MA), glycopeptides (J01XA), polymyxins (J01XB), daptomycin (J01XX09) and oxazolidinones: linezolid (J01XX08) and tedizolid (J01XX11) [15].

### Statistical analysis

Adjustment for design effect due to clustering of antimicrobial use in the participating hospitals for estimation of the confidence intervals was performed with the ‘survey’ package (v. 3.33–2) for analysis of complex survey samples in R.

For the calculation of the EU/EEA prevalence of antimicrobial use, the participating countries’ prevalence was weighted using the number of occupied beds per day as estimated by the latest available Eurostat data [16].

For countries applying the standard protocol, a multiple logistic regression model was built to predict the country prevalence of patients receiving one or more antimicrobial agents on the day of survey based on case-mix. The variables included in the model were age, sex, length of hospital stay (i.e. number of days up to the day of survey), McCabe score, intubation, presence of urinary catheter, surgery since admission, patient/consultant specialty, hospital type and hospital size [1].

For countries applying the ‘light’ protocol, and thus only submitting aggregated denominator data, the model included only patient/consultant specialty, hospital type and hospital size.

### Ethics statement

Ethical approval was at the discretion of each national public health and government body. All data shared with ECDC on patient and institutional level were anonymous.

## Results

In total, 1,753 hospitals from 29 countries participated in the PPS, of which two countries, Germany and Norway, provided aggregated denominator data on a ward level. The representativeness of the sample was optimal in 17 countries, good in 10 countries and poor in two countries (Bulgaria and the Netherlands). After adjustment for over-representation of countries contributing to the PPS with more than 20,000 patients, 325,737 patients from 1,275 hospitals remained in the dataset used for this analysis.

Pooled results were only reported for the EU/EEA corresponding to 310,755 patients from 1,209 hospitals. Of these, 357 (29.5%) were primary care hospitals, 414 (34.2%) were secondary care hospitals, 245 (20.3%) were tertiary care hospitals and 165 (13.6%) were specialised hospitals. The hospital type was unknown for 28 (2.3%) hospitals.

### Prevalence of antimicrobial use

Among all patients, 102,093 (32.9%) received at least one antimicrobial agent. Among these, 72,094 (70.6%) received one antimicrobial agent, 24,091 (23.6%) received two, 4,631 (4.5%) received three, and 1,277 (1.3%) received four or more antimicrobial agents (maximum eight). In total, 139,609 prescribed antimicrobial agents were recorded. The overall weighted prevalence of antimicrobial use in EU/EEA countries was 30.5% (range 15.9–55.6%) (Table 1). Antimicrobials for systemic use (J01) accounted for 128,881 (92.3%) prescriptions, antimycotics for systemic use (J02) for 4,425 (3.2%), antimycobacterials (J04) as second-line treatment of e.g. MRSA infections (rifampicin) or for treatment of mycobacteria other than tuberculosis (MOTT) for 2,315 (1.7%), nitroimidazole-derived antiprotozoals (P01AB) for 2,113 (1.5%), intestinal antiinfectives (A07AA) for 1,857 (1.3%) and dermatological antifungals for systemic use (D01BA) for 18 (1.3%). Most antimicrobial agents (101,638 prescriptions, 72.8%) were administered parenterally, 37,530 (26.9%) orally, 266 (0.2%) by inhalation, and 175 (0.1%) by other routes. The reason for prescribing the antimicrobial was documented in the patient’s medical records for 112,033 (80.2%) prescriptions.

**Table 1:** Prevalence of antimicrobial use, structure and process indicators of antimicrobial stewardship, by country, 28 European Union/European Economic Area countries^a^ and Serbia, 2016–2017

Country	Number of hospitals	Number of eligible patients	Antimicrobial use				Antimicrobial stewardship consultant in the hospital			Formal procedure for post-prescription review in the hospital^b^		Participation in a national or regional hospital antimicrobial consumption surveillance network	
			Number of patients with at least one antimicrobial	Observed prevalence % (95% CI)	Predicted prevalence %	DDD per 100 patients	Total number replied	Mean FTE per 250 beds	Median FTE per 250 beds	Total number replied	Number with procedure	Total number replied	Number with participation
**Austria**	49	13,461	3,663	27.2 (24.3–30.2)	31.9	40.3	49	0.14	0	49	31	9	9
**Belgium**	43	11,800	3,320	28.1 (26.6–29.7)	30.2	45.5	35	0.33	0.23	41	18	25	18
**Bulgaria**	12	2,200	995	45.2 (39.8–50.3)	38.7	54.3	12	0.63	0.50	11	9	3	2
**Croatia**	34	10,466	3,263	31.2 (26.6–35.8)	33.8	42.0	31	0.60	0	34	12	25	20
**Cyprus**	8	1,036	475	45.8 (42.9–48.8)	42.3	70.6	8	0	0	8	1	5	0
**Czech Republic**	45	15,117	4,386	29.0 (27.2–30.8)	36.9	48.1	45	0.49	0.28	5	2	45	0
**Estonia**	23	4,220	1,059	25.1 (21.2–29.0)	29.6	38.0	14	0.13	0.13	20	11	15	2
**Finland**	51	9,079	3,485	38.4 (35.0–41.7)	34.8	49.8	35	0.28	0.08	46	23	9	9
**France**	50	16,522	3,259	19.7 (17.9–21.5)	26.6	26.5	50	0.67	0.25	50	46	50	44
**Germany**	49	11,324	2,437	21.5 (17.2–25.8)	28.2	31.8	46	0.14	0	49	12	49	16
**Greece**	42	9,401	5,227	55.6 (53.1–58.1)	42.1	N	27	0.14	0.09	27	18	36	18
**Hungary**	38	20,588	3,282	15.9 (13.2–18.6)	23.9	19.8	38	0.16	0	35	5	8	8
**Iceland**	2	633	190	30.0 (28.5–31.5)	28.3	35.4	2	0	0	2	0	1	0
**Ireland**	60	10,333	4,104	39.7 (37.4–42.0)	35.2	68.2	56	0.54	0.60	58	43	60	46
**Italy**	56	14,773	6,579	44.5 (42.6–46.5)	40.0	64.6	55	0.42	0	55	21	53	20
**Latvia**	14	3,807	1,459	38.3 (35.1–41.6)	34.7	51.0	11	0.11	0	14	2	14	1
**Lithuania**	62	12,415	3,370	27.1 (23.9–30.4)	26.6	37.9	60	0.35	0	61	34	62	60
**Luxembourg**	12	2,018	516	25.6 (19.4–31.7)	27.7	39.8	12	0.71	0	12	3	9	7
**Malta**	4	961	385	40.1 (37.8–42.4)	35.1	64.8	4	0.16	0	4	1	4	1
**The Netherlands**	19	4,441	1,471	33.1 (31.5–34.7)	37.8	49.7	7	0.03	0	4	3	12	10
**Norway**	43	9,628	2,868	29.8 (28.0–31.4)	34.7	55.0	24	0.22	0.08	24	18	24	24
**Poland**	80	21,712	6,073	28.0 (25.7–30.2)	33.4	36.7	80	0.16	0.07	79	32	43	4
**Portugal**	93	16,982	6,722	39.6 (36.9–42.3)	37.2	51.7	81	0.22	0	93	37	60	38
**Romania**	40	11,443	4,829	42.2 (38.7–45.7)	35.4	53.7	36	0.54	0.24	40	27	36	34
**Slovakia**	50	9,145	2,641	28.9 (26.2–31.6)	30.2	42.6	46	0.50	0	50	32	29	4
**Slovenia**	20	5,720	1,787	31.2 (28.8–33.7)	37.4	45.3	20	0.07	0	20	3	20	12
**Spain**	96	19,546	9,054	46.3 (44.8–47.9)	39.3	66.4	80	0.46	0.12	72	29	78	30
**UK – England**	32	20,148	7,533	37.4 (35.3–39.5)	35.2	64.2	32	0.58	0.45	32	32	32	32
**UK – Northern Ireland**	16	3,813	1,385	36.3 (32.3–40.3)	36.6	68.8	16	0.53	0.55	16	14	16	16
**UK – Scotland**	45	11,623	4,093	35.2 (33.3–37.1)	35.1	69.2	42	0.58	0.29	45	28	45	39
**UK – Wales**	21	6,400	2,186	34.2 (32.0–36.4)	34.5	56.9	21	0.75	0.32	19	17	21	17
**EU/EEA**	**1 209**	**310,755**	**102,093**	**30.5 (29.2–31.9)^c^**	**NA**	**46.0**	**1,075**	**0.37**	**0.08**	**1,075**	**564**	**898**	**541**
**Serbia**	66	14,982	6,185	41.3 (38.9–43.7)	36.9	53.1	61	0.32	0	66	24	8	7

CI: confidence interval; DDD: defined daily dose; EU/EEA: European Union/European Economic Area; FTE: full-time equivalent; N: not available; NA: not applicable; UK: United Kingdom.

^a^For the UK, data for England, Northern Ireland, Scotland and Wales are presented separately.

^b^Review of the appropriateness of prescribed antimicrobials within 72 hours (three calendar days) from the initial order, in at least one of the hospital wards.

^c^Observed prevalence is weighted.

The three EU/EEA countries that did not participate were Denmark, Lichtenstein and Sweden.

### Indications for antimicrobial use

**Figure 1:**
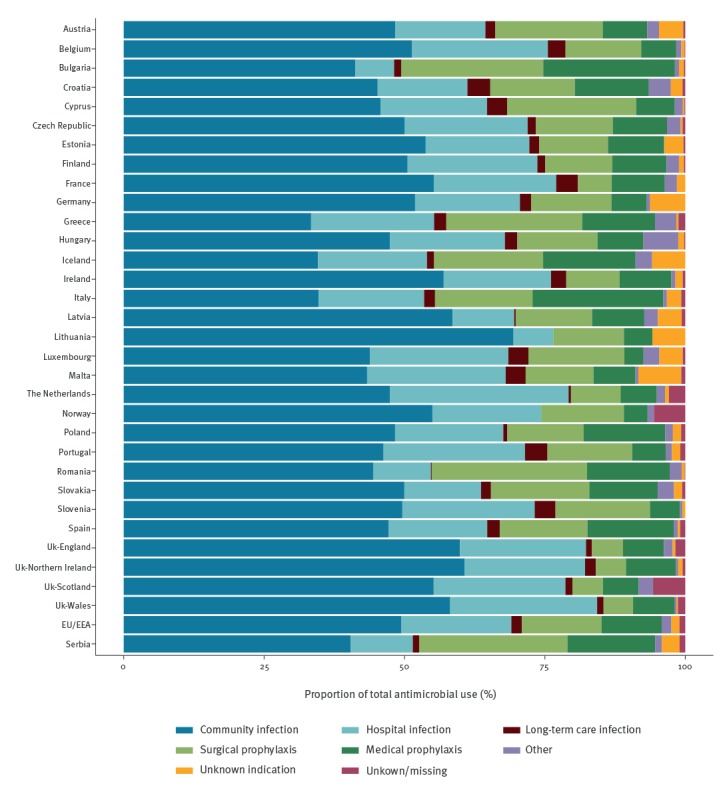
Indications for antimicrobial use in acute care hospitals, 28 European Union/European Economic Area countries^a^ and Serbia, 2016–2017

Of 139,609 antimicrobial agents prescribed, 98,986 (70.9%) were for treatment of infection and of these 69.8% were prescribed for the treatment of a community-acquired infection (Figure 1). The most common site of infection was the respiratory tract (31.8%), followed by systemic infections (14.7%), the urinary tract (13.9%) and the gastrointestinal tract (13.6%). Other body sites accounted for 26.0% of the site of infection for antimicrobial treatment. 

UK: United Kingdom.

^a^For the UK, data for England, Northern Ireland, Scotland and Wales are presented separately.

The three EU/EEA countries that did not participate were Denmark, Lichtenstein and Sweden.

**Figure 2:**
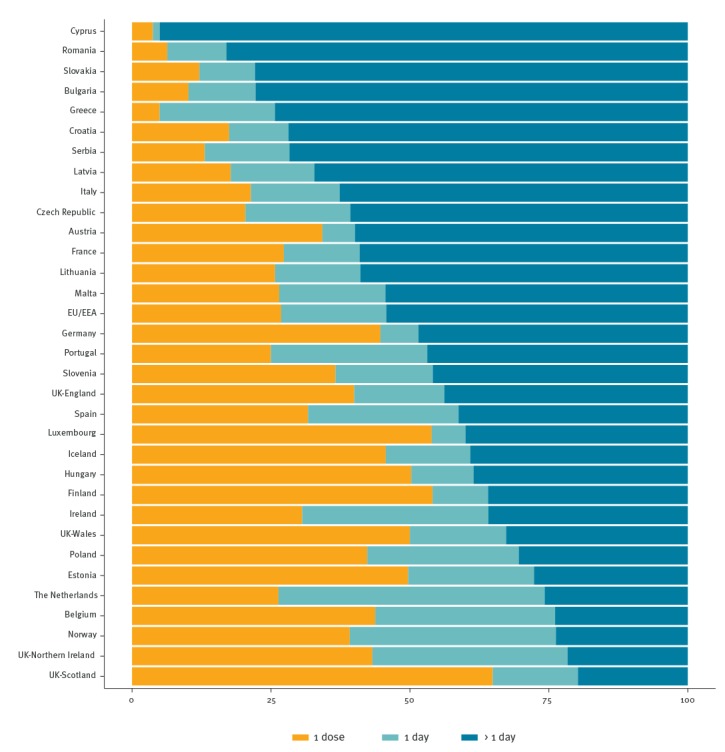
Surgical prophylaxis in acute care hospitals, by dose and duration, 28 European Union/European Economic Area countries^a^ and Serbia, 2016–2017

The proportion of antimicrobial agents prescribed for prophylaxis was 24.9%. More than half (10,741/19,798, 54.2%) of surgical prophylaxis courses were prescribed for more than 1 day (country range 19.8–95.0%) (Figure 2).

UK: United Kingdom.

^a^For the UK, data for England, Northern Ireland, Scotland and Wales are presented separately.

The three EU/EEA counties that did not participate were Denmark, Lichtenstein and Sweden.

### Most commonly used antimicrobial agents

**Figure 3:**
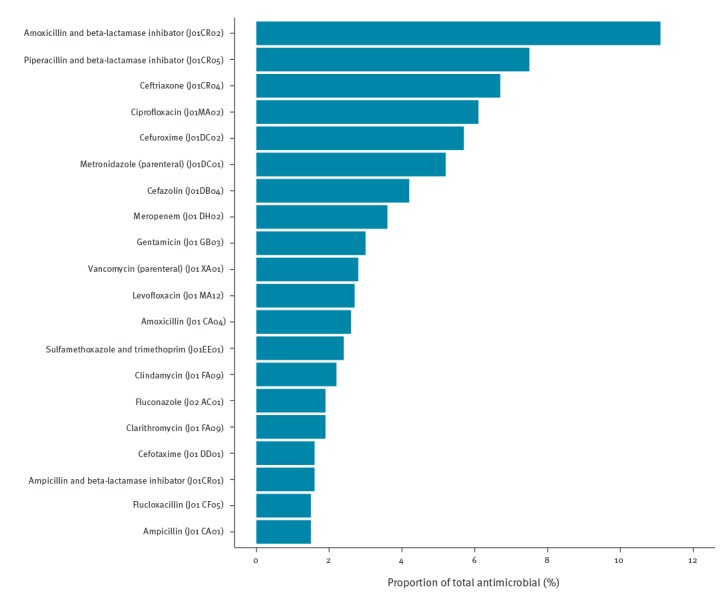
Antimicrobial agents (ATC code) accounting for 75% of antimicrobial use (Drug Utilisation 75%) in acute care hospitals, European Union/European Economic Area countries, 2016–2017

The antimicrobial agents that accounted for 75% of total antimicrobial use (DU75%) are presented in Figure 3. Antimicrobial prescription varied by indication. Of 27,324 antimicrobial prescriptions used for the treatment of HAIs, combination of penicillins with beta-lactamase inhibitors (J01CR) were the antimicrobial agents most commonly used (19.8%) followed by carbapenems (J01DH) and fluoroquinolones (J01MA) with 9.9% and 9.4%, respectively.

ATC: Anatomical Therapeutic Chemical.

The three EU/EEA counties that did not participate were Denmark, Lichtenstein and Sweden.

Of 69,067 antimicrobial prescriptions for the treatment of community-acquired infections, the three antimicrobial agents most commonly prescribed were combinations of penicillins and beta-lactamase inhibitors (J01CR: mainly amoxicillin and beta-lactamase inhibitor, J01CR02, and piperacillin and beta-lactamase inhibitor, J01CR05) followed by third-generation cephalosporins (J01DD) and fluoroquinolones (J01MA) with 23.2%, 11.7% and 11.1%, respectively.

**Figure 4:**
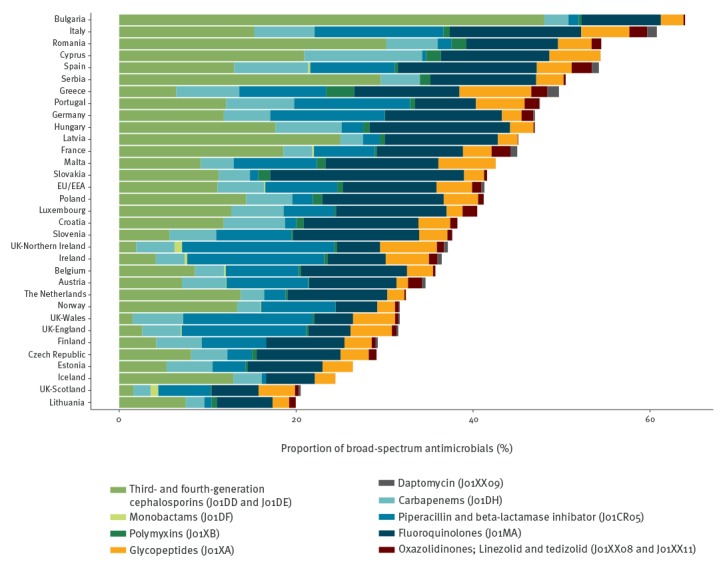
Proportion of broad-spectrum antibacterials^a^ among all antibacterials for systemic use (J01), 28 European Union/European Economic Area countries^b^ and Serbia, 2016–2017

Of 19,798 antimicrobial prescriptions for surgical prophylaxis, the three most common antimicrobial agents were first-generation cephalosporins (J01DB), second-generation cephalosporins (J01DC) and combinations of penicillins with beta-lactamase inhibitors (J01CR), with 26.6%, 17.9% and 15.1%, respectively. The proportion of broad-spectrum antibacterials among all antibacterials for systemic use (J01) is shown in Figure 4.

UK: United Kingdom.

^a^As defined in the European Centre for Disease Prevention and Control, European Food Safety Authority and European Medicines Agency Joint Scientific Opinion: piperacillin and beta-lactamase inhibitor (ATC J01CR05), third- and fourth-generation cephalosporins (J01DD and J01DE), monobactams (J01DF), carbapenems (J01DH), fluoroquinolones (J01MA), glycopeptides (J01XA), polymyxins (J01XB), daptomycin (J01XX09) and oxazolidinones: linezolid (J01XX08) and tedizolid (J01XX11) [15].

^b^For the UK, data for England, Northern Ireland, Scotland and Wales are presented separately.

The three EU/EEA counties that did not participate were Denmark, Lichtenstein and Sweden.

### Change of antimicrobial agent

**Figure 5:**
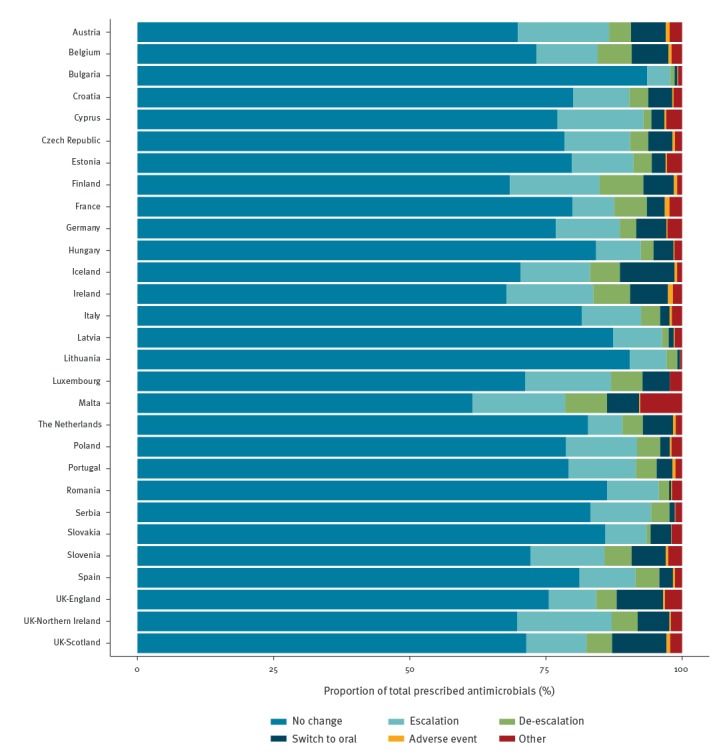
Change of antimicrobial during the infection episode and reported reason for change, 26 European Union/European Economic Area countries^a^ and Serbia, 2016–2017

In total, information about change of the antimicrobial during the infection episode was reported for 76.8% of antimicrobial prescriptions. For antimicrobial prescriptions where the information was reported, most (79.0%, country range: 61.5–93.6%) had not been changed since the initiation of the treatment (Figure 5). Escalation, de-escalation and switch from intravenous to oral use were reported for 10.9%, 3.9%, and 4.0% antimicrobial prescriptions, respectively. The change was due to adverse effects for 0.4% and to other reasons for 1.8% prescriptions.

UK: United Kingdom.

^a^For the UK, data for England, Northern Ireland and Scotland are presented separately.

Greece, Norway and UK-Wales did not collect information on change of antimicrobials.

The three EU/EEA counties that did not participate were Denmark, Lichtenstein and Sweden.

### Antimicrobial stewardship structure and process indicators

The median full-time equivalents for antimicrobial stewardship consultants per 250 beds was 0.08 (country range: 0–0.60), with 76.3% of the participating hospitals reporting antimicrobial use guidelines and 54.3% reporting some dedicated time for antimicrobial stewardship. Among the hospitals that submitted information on structure and process indicators for antimicrobial stewardship, the proportion of hospitals in the EU/EEA participating countries that had implemented a formal policy for post-prescription review in at least one ward was 52.5% while the proportion of hospitals participating in a national or regional hospital antimicrobial consumption surveillance network was 60.2% (Table 1).

## Discussion

One in three patients hospitalised in acute care hospitals in the EU/EEA received one or more antimicrobials on the day of the PPS. The majority of the antimicrobials were prescribed for the treatment of a community-acquired infection. However, almost one in five antimicrobial prescriptions was for the treatment of a HAI. Prevention and control of HAIs reduces the need for antimicrobials and is an essential component of strategies to reduce unnecessary antimicrobial use. Antimicrobial use was similar to or lower than what was observed in other studies, such as the international PPS (range: 27.4–50.0%) [17] or the United States (US) 2011 PPS (49.9%) [18].

About one in seven antimicrobial prescriptions was for surgical prophylaxis, which represented the third most common indication. Surgical prophylaxis is recommended for the prevention of surgical site infections [19,20]. For the majority of surgical procedures, one preoperative dose is sufficient. In this PPS, however, more than half of the antimicrobial courses for surgical prophylaxis lasted more than 1 day. Although this proportion slightly decreased since the first survey in 2011–12 (54% vs 59%), it remains very high and outside the recommended duration in common with other studies where it ranged from 40.6% to 86.3% [17]. This is an important source of unnecessary use of antimicrobials and should be a priority target for future efforts on antimicrobial stewardship in many European acute care hospitals.

Overall, more than one in 10 antimicrobial prescriptions were for medical prophylaxis. This proportion is higher than the proportion of medical prophylaxis in the international PPS (7.4%) [17] and the proportion of medical prophylaxis in the US 2011 PPS (6.9%) [18]. Given the limited number of indications for medical prophylaxis and that it should only be used when indicated in relevant guidelines [9], a proportion of these prescriptions may represent antimicrobial use without clear indication and are therefore, unnecessary.

Pneumonia was by far the most common indication for antimicrobial treatment, accounting for one in four antimicrobials prescribed for therapeutic indications. Lower urinary tract infection was the second most frequent indication, accounting for almost one in 10 prescribed antimicrobials for therapeutic indications. These results are comparable with those of the 2011–12 survey (where 23.1% of prescriptions for therapeutic indications were for pneumonia and 11.1% for lower urinary tract infection) and in line with the US 2011 PPS on antimicrobial use [18], although the proportion of antimicrobials for treatment of a urinary tract infection was slightly lower in the international PPS than in our survey [17].

There was considerable variability in the prevalence of antimicrobial use among participating countries. Although part of this variability may be explained by differences in patient case-mix and the incidence of HAIs, it also reflects differences in antimicrobial prescription practices in acute care hospitals e.g. variation in the ratio between penicillins vs other beta-lactam antibiotics (including cephalosporins and carbapenems) and fluoroquinolones between participating countries (data not shown).

The most commonly prescribed antimicrobial agents were amoxicillin and beta-lactamase inhibitor, piperacillin and beta-lactamase inhibitor and ceftriaxone. Despite extensive global shortage in 2017 [21], piperacillin and beta-lactamase inhibitor was the second most commonly used antimicrobial whereas it ranked fifth in the 2011–12 survey. By contrast, ciprofloxacin, which was the second most commonly prescribed antimicrobial agent in the 2011–12 survey, ranked fourth in 2016–17. This decrease may reflect the antimicrobial stewardship efforts or focused attempts to reduce *Clostridium difficile* infections. Fluoroquinolone and glycopeptide use was lower in the EU/EEA in 2016–17 than reported in the US 2011 PPS where these antimicrobials were the first and second most commonly prescribed ones (accounting for 14.4% and 10.8% of prescriptions, respectively) [18].

Among the reasons for change of antimicrobial during the infection episode, the proportion of de-escalation and switch from intravenous to oral administration varied among participating countries. In several countries, de-escalation or switch to oral treatment was uncommon. It was not possible to assess the appropriateness of low proportions of change, as no information was collected about the reasons for continuing or changing antimicrobial. However, both de-escalation and switch to oral treatment likely reflect the result of review of antimicrobial treatment when microbiological information is available, or when the condition of the patient improves, and are recommended measures to support prudent use of antimicrobials [9,22].

There was large variability among participating countries in the human resources available for antimicrobial stewardship as well as in the implemented antimicrobial stewardship strategies. For almost all participating countries, some hospitals had a consultant in charge of antimicrobial stewardship and while this is encouraging, considering that the majority of hospitals still have no or limited dedicated staff for antimicrobial stewardship (or access to such a consultant), promoting this must be a priority in the coming years.

In this PPS, the proportion of broad-spectrum antibacterials among all antibacterials for systemic use, as proposed by the ECDC, EFSA and EMA Joint Scientific Opinion, reflects their level of consumption in hospitals and the corresponding selection pressure [15]. These antibacterials can be found in the ‘Watch’ and ‘Reserve’ groups of antimicrobials, as defined in the WHO Model Lists of Essential Medicines [23]. In this PPS, the proportion of broad-spectrum antibacterials ranged from less than 20% to more than 50% depending on the country. This could in part be explained by the high prevalence of resistance among a number of reported microorganisms, e.g. MRSA, vancomycin-resistant enterococci or third-generation cephalosporin-resistant Enterobacteriaceae [24]. However, many of these antibacterials are also associated with both emergence and spread of healthcare-associated *Clostridium difficile* and multidrug-resistant microorganisms and in particular for third-generation cephalosporins, fluoroquinolones and carbapenems, with the emergence of multidrug-resistant Gram-negative bacteria [7], which are currently among the most important public health threats related to AMR. The wide variation and sometimes extensive use of broad-spectrum antibacterials indicates the need to review their indications in many countries and hospitals. Antimicrobial stewardship programmes must be designed to take into account both the risk of emergence of AMR and patient safety. Ensuring that broad-spectrum antibacterials are used appropriately is a key element of any strategy against AMR.

An important indicator of the quality of antimicrobial prescription is the documentation of the reason for the prescription in the patient notes. In our survey, almost one in five antimicrobial prescriptions did not include documentation of the reason for antimicrobial prescription. While this was lower than in the 2011–12 survey, it still indicates that ensuring that antimicrobial prescriptions can be reviewed effectively in all cases to assess their appropriateness remains an ongoing challenge. In the US 2011 PPS, the rationale for the antimicrobial prescription was missing only in 6.9% of prescriptions [18].

The strengths of this survey are its large size and the use of a standardised protocol across all participating hospitals in 28 EU/EEA countries and Serbia. With only two EU/EEA countries (Bulgaria and the Netherlands) having provided data on a non-representative sample of acute care hospitals and two additional EU/EEA countries (Denmark and Sweden) having declined participation, we believe that this PPS offers a representative picture of antimicrobial consumption in acute care hospitals in the EU/EEA, with meaningful benchmarks for participating countries and hospitals. The results were largely comparable to those of the 2011–12 PPS, which is both reassuring in terms of methodology but disappointing in terms of little change of antimicrobial prescription practice in European acute care hospitals in the past 5 years.

One limitation of this survey is its cross-sectional design, which evaluated antimicrobial use on 1 day only. However, this design has been shown to provide reliable results that can be used for identifying targets for intervention [2]. Moreover, the size and representativeness of the sample counterbalance this limitation. Another limitation is that we were not able to assess whether antimicrobial prescription was in line with existing international or national guidelines. However, observations such as prolonged duration of surgical prophylaxis as well as the high use of fluoroquinolones, third-generation cephalosporins and carbapenems, likely indicate inappropriate antimicrobial use that can be addressed by specific actions.

In conclusion, this second ECDC PPS of HAIs and antimicrobial use provided representative data on antimicrobial use in acute care hospitals across EU/EEA countries. These data allow for identifying targets for future antimicrobial stewardship interventions. Ultimately, these results will be helpful to promote prudent use of antimicrobials at national and European level and contribute to the efforts to ensure that European patients are receiving appropriate treatment while at the same time minimising the risk of adverse effects, and the emergence and spread of AMR.

## Notes

**Members of the Point Prevalence Survey Study Group:** Reinhild Strauss, (Federal Ministry for Labour, Social Security, Health and Consumer Protection (BMAGSK) Vienna, Austria); Elisabeth Presterl (Medical University of Vienna, Vienna, Austria); Katrien Latour (Sciensano, Brussels, Belgium); Eline Vandael (Sciensano, Brussels, Belgium); Elina Dobreva (National Center of Infectious and Parasitic Diseases (NCIPD), Sofia, Bulgaria); Ivan N. Ivanov (National Center of Infectious and Parasitic Diseases (NCIPD), Sofia, Bulgaria); Ana Budimir (University of Zagreb School of Medicine, University Hospital Centre Zagreb, Zagreb, Croatia); Zrinka Bošnjak (University of Zagreb School of Medicine, University Hospital Centre Zagreb, Zagreb, Croatia); Linos Hadjihannas (Ministry of Health, Nicosia, Cyprus); Vlastimil Jindrák (National Institute of Public Health, Prague, Czech Republic); Pille Märtin (West Tallinn Central Hospital, Health Board, Tallinn, Estonia); Piret Mitt (Tartu University Hospital, Tartu, Estonia); Outi Lyytikäinen (National Institute for Health and Welfare, Helsinki, Finland); Emmi Sarvikivi (National Institute for Health and Welfare, Helsinki, Finland); Côme Daniau (National Public Health Agency (Santé publique France), Saint Maurice, France); Anne Berger-Carbonne (National Public Health Agency (Santé publique France), Saint Maurice, France); Seven Aghdassi  (Institute of Hygiene and Environmental Medicine, Charité – University Medicine Berlin, Berlin, Germany); Petra Gastmeier (Institute of Hygiene and Environmental Medicine, Charité – University Medicine Berlin, Berlin, Germany); Flora Kontopidou (Hellenic Center for Disease Control and Prevention, Athens, Greece); Kostoula Arvaniti (Antibiotic Committee and Infection Control Unit, Papageorgiou University-affiliated Hospital, Thessaloniki, Greece); Agnes Hajdu (Department of Hospital Hygiene and Epidemiological Surveillance, Ministry of Human Capacities, Budapest, Hungary); Ólafur Guðlaugsson (Landspitali University Hospital, Reykjavik, Iceland); Carla M. Zotti (Dept. Public Health Sciences and Paediatrics; Turin, Italy); Francesca Quattrocolo (Dept. Public Health Sciences and Paediatrics; Turin, Italy); Karen Burns (Health Protection Surveillance Centre, Dublin, Ireland); Elīna Dimiņa (Centre for Disease Prevention and Control, Riga Latvia); Aija Vilde (Pauls Stradins Clinical University Hospital, Riga, Latvia); Justė Staniulytė (Institute of Hygiene (HI), Vilnius, Lithuania); Martine Debacker (Ministère de la santé - Direction de la santé, Luxembourg, Luxembourg); Vic Arendt (Centre Hospitalier de Luxembourg (CHL), Luxembourg, Luxembourg); Michael A. Borg (Mater Dei Hospital, Msida, Malta); Titia Hopmans (Centre for Infectious Disease Control Netherlands, Bilthoven, the Netherlands); Emma Smid (Centre for Infectious Disease Control Netherlands, Bilthoven, the Netherlands); Thale C. Berg (Norwegian Institute of Public Health, Oslo, Norway); Torunn Alberg (Norwegian Institute of Public Health, Oslo, Norway); Aleksander Deptuła (Nicolaus Copernicus University, Toruń; Ludwik Rydygier Collegium Medicum; Bydgoszcz, Poland); Isabel Neves (Direção-Geral da Saúde (DGS), Lisbon, Portugal); Pedro Pacheco (Direção-Geral da Saúde (DGS), Lisbon, Portugal); Roxana Ioana Serban (National Institute of Public Health (NIPH), Bucharest, Romania); Andreea Sorina Niculcea (National Institute of Public Health (NIPH), Bucharest, Romania); Ljiljana Markovic-Denic (University of Belgrade, Faculty of Medicine, Belgrade, Serbia); Gorana Dragovac (Institute of Public Health of Vojvodina; University of Novi Sad, Faculty of Medicine, Novi Sad, Serbia); Slavka Litvová (Regional Public Health Authority, Trenčín, Slovakia); Mária Štefkovičová (Alexander Dubcek University, Trenčín, and Regional Public Health Authority, Trenčín, Slovakia); Tatjana Lejko Zupanc (University Medical Center Ljubljana, Ljubljana, Slovenia); Aleš Korošec (National Institute of Public Health, Ljubljana, Slovenia); Angel Asensio (Hospital Universitario Puerta de Hierro-Majadahonda, Madrid, Spain); Mireia Cantero (Hospital Universitario Puerta de Hierro-Majadahonda, Madrid, Spain); Alan Johnson (Public Health England, London, UK); Muhammad Sartaj (Public Health Agency, Belfast, Northern Ireland, UK); Mark McConaghy (Public Health Agency, Belfast, Northern Ireland, UK); Shona Cairns (National Services Scotland, Health Protection Scotland, Glasgow, Scotland, UK); Cheryl Gibbons (National Services Scotland, Health Protection Scotland, Glasgow, Scotland, UK); Wendy Harrison (Public Health Wales, Cardiff, Wales, UK); David Florentin (Public Health Wales, Cardiff, Wales, UK).
