# Authors’ correction for Euro Surveill. 2018;23(46)

**DOI:** 10.2807/1560-7917.ES.2018.23.47.1811222

**Published:** 2018-11-22

**Authors:** Eurosurveillance editorial team

**Affiliations:** 1 European Centre for Disease Prevention and Control (ECDC), Stockholm, Sweden

As requested by the authors of the article ‘Antimicrobial use in European acute care hospitals: results from the second point prevalence survey (PPS) of healthcare-associated infections and antimicrobial use, 2016 to 2017’, published on 15 November 2018, in the Results, under the subtitle 'Prevalence of antimicrobial use,' a sentence mistakenly stated 'Antimicrobials for systemic use (J01)' instead of ‘Antibacterials for systemic use (J01)’. In the same sentence, the percentage for ‘dermatological antifungals for systemic use (D01BA) for 18 (1.3%)’ mistakenly stated ‘(1.3%)’ instead of ‘(0.01%)’. The corrections were made on 21 November 2018, as requested by the authors.

